# NOX-like ROS production by glutathione reductase

**DOI:** 10.1016/j.isci.2022.105093

**Published:** 2022-09-08

**Authors:** Julia M. Diaz, Xinying Shi

**Affiliations:** 1 Geosciences Research Division, Scripps Institution of Oceanography, University of California San Diego, La Jolla, CA 92093, USA

**Keywords:** Earth sciences, Oceanography, Microbiology, Biocatalysis, Bioengineering

## Abstract

In organisms from bacteria to mammals, NADPH oxidase (NOX) catalyzes the production of beneficial reactive oxygen species (ROS) such as superoxide (O_2_^−^). However, our previous research implicated glutathione reductase (GR), a canonical antioxidant enzyme, as a source of extracellular superoxide in the marine diatom *Thalassiosira oceanica*. Here, we expressed and characterized the two GR isoforms of *T. oceanica*. Both coupled the oxidation of NADPH, the native electron donor, to oxygen reduction, giving rise to superoxide in the absence of glutathione disulfide, the native electron acceptor. Superoxide production by ToGR1 exhibited similar kinetics as representative NOX enzymes, and inhibition assays agreed with prior organismal studies, supporting a physiological role. ToGR is similar to GR from human, yeast, and bacteria, suggesting that NOX-like ROS production by GR could be widespread. Yet unlike NOX, GR-mediated ROS production is independent of iron, which may provide an advantageous way of making ROS under micronutrient stress.

## Highlights

• Glutathione reductase expressed from a microbial phototroph is found to produce ROS
• This promiscuous reaction shows similar kinetics and inhibition as NOX-derived ROS
• The GR pathway of ROS production has cellular benefits under physiological stress
• GR may function similarly to NOX in other taxa, providing metabolic versatility

## Graphical abstract

**Figure:**
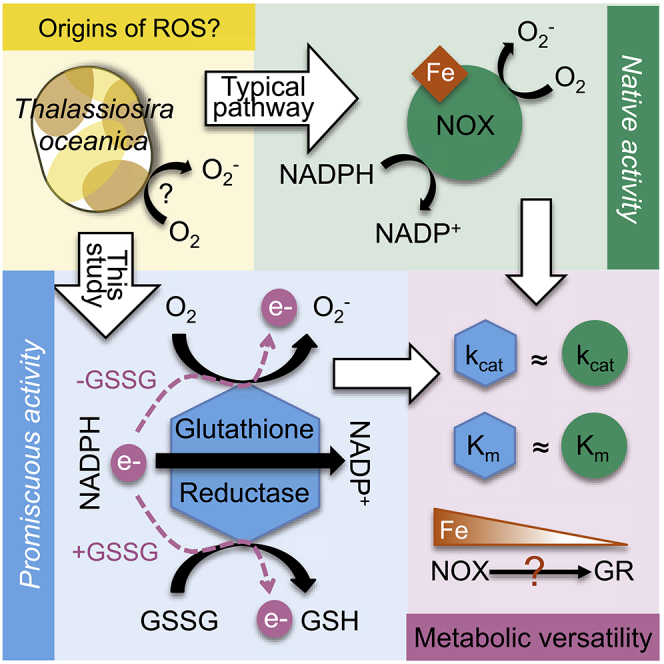

## Introduction

Superoxide is a reactive oxygen species (ROS) formed by the one-electron reduction of oxygen. All aerobic forms of life generate superoxide and other ROS, which can accumulate to toxic levels under adverse conditions. Yet physiological levels of ROS serve a broad diversity of beneficial signaling roles, as well. In organisms from bacteria to humans, the beneficial roles of ROS include growth regulation (Hansel et al., 2019; Huang et al., 2019; Patel et al., 2018; Rossi et al., 2017), innate immunity (Bardaweel et al., 2018; Weinberger, 2007), and defense (Armoza-Zvuloni et al., 2016; Diaz and Plummer, 2018; Minibayeva et al., 2009). In the ocean, the production of extracellular superoxide by microbes is widespread (Diaz et al., 2013; Diaz and Plummer, 2018; Hansel and Diaz, 2021; Sutherland et al., 2019), but the mechanisms and physiological functions of this microbial ROS production are not completely understood. Addressing this knowledge gap will improve our basic understanding of microbial ecosystem services and ocean health under continued global change.

Phytoplankton are photosynthetic microbes in the ocean that form the base of marine food webs, contribute to global climate by absorbing the greenhouse gas carbon dioxide, and produce at least half of the world’s oxygen supply. In a previous study, we investigated the mechanism and role of extracellular superoxide production by the phytoplankton species *Thalassiosira oceanica*, which represents the diatom lineage of marine phytoplankton (Malviya et al., 2016), a dominant group responsible for approximately 20% of global photosynthesis (Falkowski et al., 1998; Field et al., 1998; Nelson et al., 1995). In *T. oceanica*, we found that NADPH-dependent extracellular superoxide production is vital to photophysiology (Diaz et al., 2019). An expected source of this ROS production would be the enzyme NADPH oxidase (NOX), which couples the oxidation of NADPH to the generation of superoxide. Indeed, since its discovery in human phagocytes, NOX has been found in bacteria (Hajjar et al., 2017; Magnani et al., 2017) and every major eukaryotic lineage (Bedard et al., 2007) and has been implicated in extracellular superoxide production by several phytoplankton taxa (Hansel and Diaz, 2021). Yet surprisingly, our previous results revealed an unexpected enzyme as a source of extracellular superoxide production by *T. oceanica*: glutathione reductase (GR) (Diaz et al., 2019).

GR is a highly conserved enzyme across the tree of life. It typically promotes a reducing environment by coupling the oxidation of NADPH to the reduction of glutathione disulfide (GSSG), yielding reduced glutathione (GSH). For example, GSH directly eliminates some ROS, with the exception of superoxide (Winterbourn, 2016), acts as a thiol buffer maintaining proteins in their reduced state, and serves as a substrate of glutathione peroxidase, which degrades the ROS hydrogen peroxide (H_2_O_2_). GR can donate electrons to a wide variety of acceptors besides the native substrate GSSG (Carlberg and Mannervik, 1986; Cénas et al., 1989; Nordman et al., 2003; Paulíková and Berczeliová, 2005; Pinto et al., 1985), including oxygen (Angiulli et al., 2015; Korge et al., 2015). ROS production by GR has been reported previously in plant (Asada, 1999), yeast (Massey et al., 1969), bovine (Liochev and Fridovich, 1992), and human (Coppo et al., 2022; Korge et al., 2015), but GR is not widely recognized as a physiological ROS source.

Here, we characterized the capacity of *T. oceanica* GR (ToGR) to produce superoxide *in vitro* in order to evaluate the potential physiological relevance of this promiscuous activity. Two putative GR isoforms exist in the *T. oceanica* genome: ToGR1 and ToGR2 (Diaz et al., 2019; Lommer et al., 2012). Both ToGR proteins possess unique ∼130 amino acid N-terminal putative localization domains that appear absent in other GRs, yet the functional ToGR proteins show high sequence similarity to each other and to other GRs from human, yeast, and bacteria, including several highly conserved sites of known catalytic or structural importance (Diaz et al., 2019). In addition, GRs share several broad features with NOX enzymes, including the ability to bind and oxidize NADPH and to transfer electrons using a flavin adenine dinucleotide (FAD) cofactor in the active site. Overall, results revealed that both ToGRs produce superoxide, and that ROS production by ToGR1 likely has a physiological role, with a catalytic performance that is similar to representative NOX enzymes.

## Results and discussion

### ToGR1 and ToGR2 are glutathione reductases

**Figure 1:**
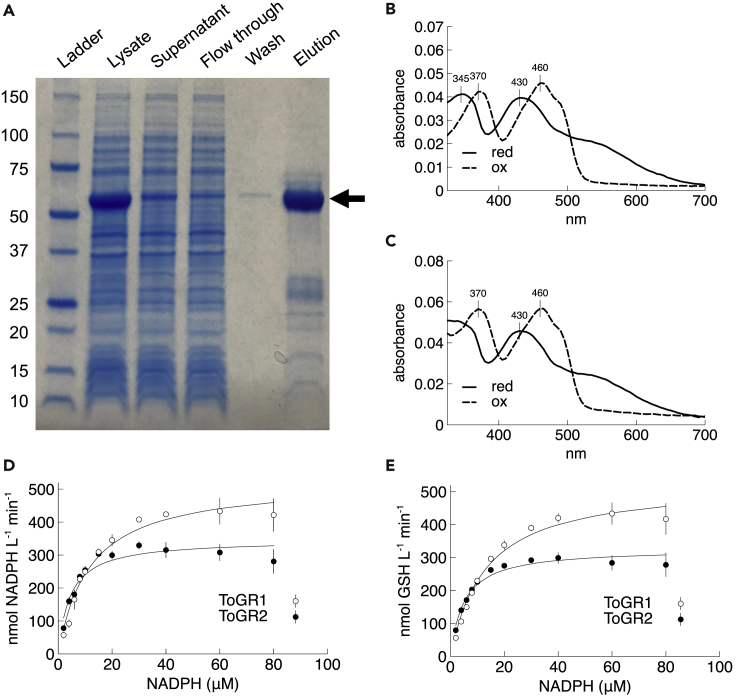
Protein properties and native activity (A) SDS-PAGE analysis of protein fractions obtained during the purification of recombinant ToGR1 (see STAR Methods). Arrow indicates ∼55 kDa. (B and C) Absorption spectra of ToGR1 (B) and ToGR2 (C) in 100 mM phosphate buffer (1 mM EDTA, pH 8.0). Proteins (10 μM) were reduced with DTT (0.1 mM) or oxidized with GSSG (1 mM). (D and E) Michaelis-Menten curves of NADPH oxidation (D) coupled to glutathione disulfide (GSSG) reduction to glutathione (GSH) (E) by ToGR1 and ToGR2 (0.045 nM enzyme concentrations) in 100 mM phosphate buffer (1 mM EDTA, pH 8.0). Rate data represent the avg. ± std. dev. of triplicate measurements, where error bars smaller than the data symbol are not visible.

We overexpressed recombinant ToGR1 and ToGR2 in *Escherichia coli* with the N-terminal localization domains removed (see STAR Methods), which yielded proteins enriched in the ∼55 kDa size fraction under denaturing conditions (Figure 1A; Figure S1). This result is consistent with the size of previously characterized GR proteins, which are homodimeric enzymes of ∼110 kDa (Deponte, 2013) that would disassociate into equivalent protein subunits no more than ∼55 kDa each. Furthermore, absorption spectra of both proteins showed peaks at 370–375 and 460 nm in oxidized form or 345 nm and 430–435 nm in reduced form (Figures 1B and 1C), which is characteristic of GR and other flavoproteins (Ji et al., 2015).

Consistent with the predicted native function of ToGR1 and ToGR2, both purified proteins coupled the oxidation of NADPH to the reduction of GSSG (Figures 1D, 1E and S2A). Kinetic analysis of the native activity revealed that ToGR1 and ToGR2 are similar to previously characterized GRs. For example, the turnover rate (k_cat_) of ToGR1 (∼12000 min^−1^) is similar to most GRs, which ranges from 12500 to 16,000 min^−1^ (Can et al., 2010; Ji et al., 2015; Mapson and Isherwood, 1963; Massey and Williams, 1965; Savvides and Karplus, 1996; Worthington and Rosemeyer, 1976). ToGR2 has a slower turnover rate than ToGR1 (∼7500 min^−1^), but it is in line with previously reported values of 8295 (López-Barea and Lee, 1979) and ∼3000 min^−1^ (Hakam and Simon, 2000).

The K_m_ values for ToGR native activity are also similar to previously characterized GRs, which typically range from 4 to 15 μM NADPH (Can et al., 2010; Hakam and Simon, 2000; Ji et al., 2015; López-Barea and Lee, 1979; Mapson and Isherwood, 1963; Massey and Williams, 1965; Worthington and Rosemeyer, 1976) (Table 1). Compared to ToGR1, ToGR2 was more readily saturated with NADPH by nearly a factor of three, making ToGR2 almost twice as specific (k_cat_ K_m_^−1^) as ToGR1 (Table 1). ToGR1 and ToGR2 specificity constants for the native activity (Table 1) are also in agreement with typical GRs (1.7×10^7^–6.6×10^7^ M^−1^s^−1^) (Can et al., 2010; Ji et al., 2015; López-Barea and Lee, 1979; Mapson and Isherwood, 1963; Massey and Williams, 1965; Worthington and Rosemeyer, 1976).

**Table 1:** Kinetic parameters of glutathione reductase activity and superoxide production by ToGR1 and ToGR2 in 100 mM phosphate buffer (pH 8.0)

Enzyme	Activity	Substrate or product	k_cat_^−1^ (min^−1^)	K_m_ (μM)	k_cat_ K_m,_^−1^ (M^−1^ s^−1^)	k_cat_/k_uncat_^a^
ToGR1	Native	NADPH	11,707 ± 671	11.7 ± 1.6	(1.7 ± 0.1)×10^7^	10^7.7^
		GSH	11,765 ± 676	13.0 ± 1.3	(1.5 ± 0.1)×10^7^	10^7.0^
	Promiscuous	NADPH	12.9 ± 1.0	23.3 ± 2.8	9626 ± 979	10^4.7^
		O_2_^−^	10.0 ± 0.8	47.5 ± 4.1	3109 ± 289	10^4.1^
ToGR2	Native	NADPH	7692 ± 312	4.4 ± 0.5	(3.0 ± 0.3)×10^7^	10^7.5^
		GSH	7273 ± 243	5.0 ± 0.4	(2.5 ± 0.1)×10^7^	10^6.8^
	Promiscuous	NADPH	1.66 ± 0.13	82.7 ± 7.3	341 ± 47	10^3.9^
		O_2_^−^	0.42 ± 0.11	210 ± 98	42 ± 11	10^2.7^

Data represent the avg. ± std. err. of n = 3 (native activities), n = 8 (ToGR1 promiscuous) or n = 5 observations (ToGR2 promiscuous). All K_m_ values are for NADPH.

a Values for k_uncat_ are listed in Table S2.

### ToGR1 and ToGR2 catalyze NADPH-dependent superoxide production

**Figure 2:**
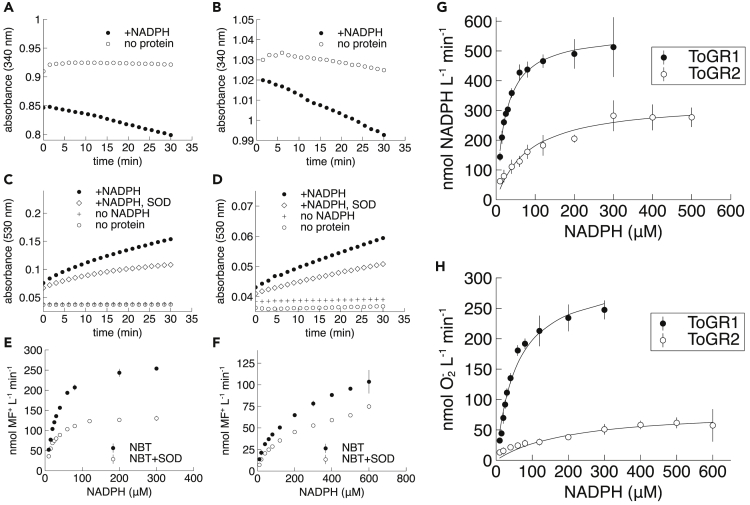
NADPH-dependent superoxide production (A–F) Absorbance of NADPH (60 μM initial concentration) at 340 nm, (C,D) simultaneous absorbance of monoformazan (MF^+^) at 530 nm, and (E,F) rates of MF^+^ production with or without superoxide dismutase (SOD). Reactions were with ToGR1 (A,C,E), ToGR2 (B,D,F), or no protein controls. (G and H) Michaelis-Menten curves of NADPH oxidation (G) coupled to the production of superoxide (H). Data reflect single representative trials (A–D) or the avg. ± std. dev. of triplicate measurements (E–H), where error bars smaller than the data symbol are not visible. All reactions were in 100 mM phosphate buffer (1 mM EDTA, pH 8.0) in the absence of GSSG. ToGR1 (40 nM), ToGR2 (200 nM).

In the absence of GSSG, the native electron acceptor, both ToGR1 and ToGR2 still oxidized NADPH, as observed by the decline in NADPH absorbance at 340 nm over time (Figures 2A and 2B). To test whether this promiscuous NADPH oxidation was coupled to superoxide production, we used the probe nitroblue tetrazolium (NBT), which is reduced to the chromogenic product monoformazan (MF^+^) in the presence of superoxide. Consistent with superoxide production, MF^+^ accumulated over time, as indicated by the increase in absorbance at 530 nm (Figures 2C and 2D).

Superoxide production is proportional to the amount of MF^+^ production inhibited by superoxide dismutase (SOD), which selectively degrades superoxide (Figures 2C–2F; Figure S2B). At most, we found that 50% and 80% of MF^+^ production by ToGR1 and ToGR2, respectively, were due to reaction of NBT with superoxide. Increasing the concentration of SOD did not alter this ratio (Figure S3), suggesting that the remainder of MF^+^ production was due to direct enzymatic reduction of NBT. A similar amount of superoxide-mediated reduction of the probe cytochrome *c* was reported in a study of bacterial NOX (Hajjar et al., 2017). Moreover, no MF^+^ formation occurred in control reactions lacking ToGR or NADPH, indicating that superoxide production was driven by NADPH-dependent enzyme activity (Figures 2C and 2D).

### ToGR1 is more specialized for superoxide production than ToGR2

Based on the concentrations obtained from Figures 2A to 2D, we calculated an MF^+^:NADPH stoichiometry of 0.70–0.98 for ToGR1 (95% confidence interval, n = 43), which approaches the ideal value of 1 (Bielski et al., 1980). However, ToGR2 only produced 0.04–0.10 molecules of MF^+^ for every molecule of NADPH oxidized (95% confidence interval, n = 18), which was significantly less than ToGR1 (p < 0.0001, Student’s *t* test). The remainder of NADPH oxidation could potentially be accounted for by the production of other reduced products, including hydrogen peroxide (Korge et al., 2015) and water (Angiulli et al., 2015), which do not reduce NBT, but can arise from NADPH-dependent oxygen reduction by GR. This result suggests that ToGR1 is more specialized for superoxide production than ToGR2.

**Figure 3:**
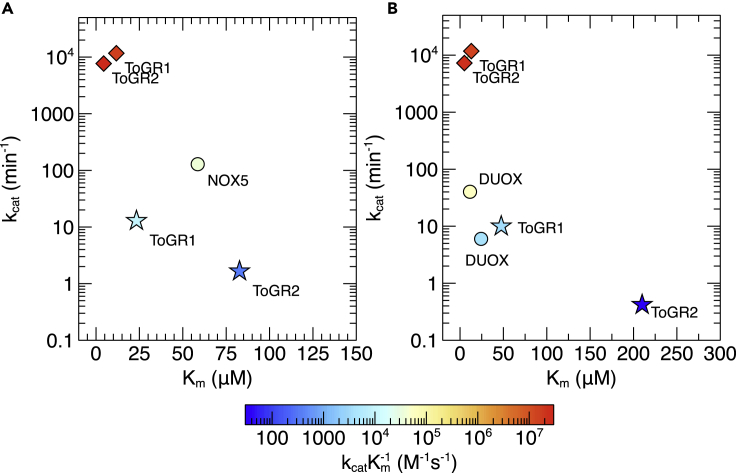
Kinetic parameters (A and B). ToGR1 and ToGR2 catalyze the oxidation of NADPH (A) and the production of glutathione or superoxide (B) in the presence (diamonds) or absence (stars) of glutathione disulfide, respectively. NOX data (circles) for NADPH oxidation (Magnani et al., 2017) and ROS production (Wu et al., 2021) are provided for reference.

Promiscuous NADPH oxidation and superoxide production followed Michaelis-Menten kinetics (Figures 2G and 2H). Overall, kinetic parameters revealed that the native GR function was superior to the promiscuous activity in both enzymes (Figure 3; Table 1). Yet promiscuous superoxide production (NADPH oxidation) by ToGR1 was ∼20 (∼8) times faster (k_cat_), ∼70 (∼30) times more specific (k_cat_ K_m_^−1^), and exhibited K_m_ values that were ∼80% (70%) lower than ToGR2 (Figure 3; Table 1). Furthermore, both enzymes enhanced the uncatalyzed rates of NADPH oxidation and superoxide production (k_uncat_), but the effect of ToGR1 was more pronounced than ToGR2 by about an order of magnitude (k_cat_ k_uncat_^−1^) (Table 1).

The differences in activity between ToGR1 and ToGR2 likely come down to three amino acid substitutions that distinguish the two functional protein sequences (Diaz et al., 2019). Two of these substitutions occur within known regions of structural or catalytic importance: the C-terminal interface-binding domain that forms the functional homodimer, and a flavin adenine dinucleotide (FAD)-binding domain (Diaz et al., 2019). However, the potential influence of these substitutions on enzyme activity remains unclear.

### Superoxide production by ToGR1 is physiologically relevant

ToGR1 may be almost as effective at catalyzing the NADPH-dependent production of ROS as some NOX enzymes (Figure 3). For example, the rate (k_cat_) of superoxide production by ToGR1 (10 min^−1^) is comparable to rates of H_2_O_2_ production by the NOX family protein dual oxidase, or DUOX (6–40 min^−1^) (Wu et al., 2021). Furthermore, the specificity constant of superoxide production (k_cat_ K_m_^−1^) by ToGR1 (3109 M^−1^s^−1^) is only 1.3 to 19 times lower than the specificity constant of H_2_O_2_ production by DUOX (4100–58000 M^−1^s^−1^). Similarly, the K_m_ of NADPH oxidation by ToGR1 (23.3 μM) is ∼2.5-fold enhanced relative to cyanobacterial NOX5 (58.6 μM), and the specificity constant of promiscuous NADPH oxidation by ToGR1 (9626 M^−1^s^−1^) is only ∼4 times lower than NOX5 (37,000 M^−1^s^−1^) (Magnani et al., 2017). These results are based on the activities of purified enzymes *in vitro*, which are likely to be different under physiological conditions. Nonetheless, these comparisons illustrate that as a source of ROS production, ToGR1 has the potential to perform similarly to representative NOX enzymes, which suggests that this promiscuous activity is physiologically relevant.

**Figure 4:**
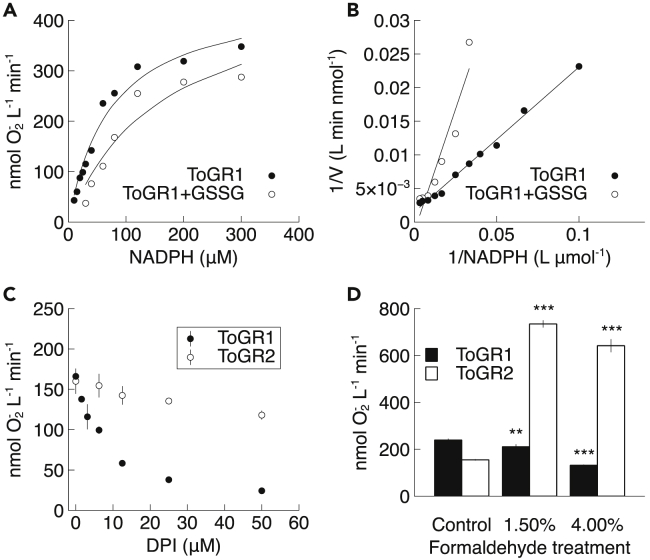
Inhibition of superoxide production (A–D). Superoxide production in the presence of (A,B) glutathione disulfide (GSSG), (C) diphenylene iodonium (DPI), or (D) after treatment of ToGR with formaldehyde. In panels c and d, data represent the avg. ± std. dev. of triplicate observations. Stars depict significant difference from the control condition (∗∗ = p < 0.001; ∗∗∗ = p < 0.0001, Student’s *t* test).

In addition to kinetic analyses, we also tested the effect of several chemical compounds on ToGR-mediated superoxide production. Some compounds eliminate *in vivo* extracellular superoxide production by *T. oceanica* and other phytoplankton, including GSSG (Diaz et al., 2019) and diphenyleneiodonium (DPI) (Anderson et al., 2016; Diaz et al., 2019; Kustka et al., 2005; Laohavisit et al., 2015; Park et al., 2009; Saragosti et al., 2010), whereas other compounds like formaldehyde do not (Schneider et al., 2016). We found that these compounds had identical effects on superoxide production by both ToGRs, but especially ToGR1, consistent with a physiological role. For example, DPI is an inhibitor of NOX enzymes and is commonly used to evaluate the potential involvement of NOX in ROS production, yet DPI can target flavoenzymes like GR, as well (Reis et al., 2020). Indeed, the application of DPI resulted in a concentration-dependent inhibition of superoxide production (Figure 4C) and NADPH oxidation (Figure S4) by ToGR1 and ToGR2. ToGR1 was more sensitive to DPI inhibition than ToGR2, with IC_50_ values that were ∼two orders of magnitude lower (Table S1). These results confirm that DPI cannot be used to distinguish between NOX and GR as potential sources of ROS production *in vivo*.

A previous study suggested that extracellular superoxide production by *Thalassiosira* spp. occurs by a passive or non-enzymatic photochemical process based on the finding that extracellular superoxide production could not be completely quenched by using formaldehyde to kill the cells (Schneider et al., 2016). However, our results show that formaldehyde failed to eliminate superoxide production by either ToGR1 or ToGR2 (Figure 4D). Therefore, we suggest that residual enzyme activity in dead cells, or enzymes interacting directly with the formaldehyde, could create the effect observed in the study by Schneider et al. (2016). In fact, formaldehyde decreased superoxide production by ToGR1 without completely abolishing it, similar to previous results from *T. oceanica* cells (Schneider et al., 2016). This result is consistent with ToGR1 as an *in vivo* source of superoxide in *T. oceanica*. On the other hand, formaldehyde stimulated superoxide production by ToGR2, which was not seen *in vivo* (Schneider et al., 2016). This disagreement suggests that ToGR2 may not be a major source of superoxide in *T. oceanica in vivo*.

Based on the observation that GSSG inhibits extracellular superoxide production by *T. oceanica*, we hypothesized previously that GR reduces oxygen and gives rise to superoxide at the GSSG binding site of the enzyme (Diaz et al., 2019). In agreement, our findings suggest that GSSG is a competitive inhibitor of superoxide production by ToGR1 (Figures 4A and 4B). Indeed, the rate constant of superoxide production (k_cat_) was similar in the absence (10.0 ± 2.4 min^−1^, n = 8) or presence (12.2 ± 0.1 min^−1^, n = 2) of GSSG, while the K_m_ of superoxide production (47.5 ± 11.4 μM, n = 8) increased by approximately 3-fold (140.7 ± 33.3 μM, n = 2) in the presence of GSSG (avg. ± std. dev.), consistent with competitive inhibition. Rates of superoxide production by ToGR2 were too low to allow a similar analysis.

A major factor controlling the ability of GR to produce superoxide under physiological conditions is GSSG. GR-mediated production of ROS is favored at low concentrations of GSSG. This process has been called ROS “spillover,” which may be triggered by reductive stress (high GSH:GSSG and NADPH:NADP^+^) and has a proposed role in redox signaling and oxidative injury in mitochondria (Korge et al., 2015). Furthermore, GR-mediated superoxide production has been implicated in multiple human disease traits (Coppo et al., 2022). While these examples highlight the harmful potential of ROS, we previously found that the production of extracellular superoxide by ToGR may benefit the photophysiological health of *T. oceanica* by alleviating reductive stress that occurs with excessive light exposure (Diaz et al., 2019). In phototrophs, extracellular superoxide production may help alleviate reductive stress that occurs under other conditions as well, including carbon dioxide limitation (Yuasa et al., 2020b) or nutrient deficiency (Yuasa et al., 2020a).

### Conclusions

In a previous study, we identified GR as a potential source of extracellular superoxide production by the marine diatom *T. oceanica* (Diaz et al., 2019). Here, we expressed, purified, and characterized the two ToGR isoforms to assess their physiological relevance. Our results confirmed that both enzymes are GRs capable of superoxide production, yet results from kinetic analyses and inhibitor assays point to ToGR1 as the likelier source of GR-mediated extracellular superoxide production *in vivo*. These findings support the view that ToGR1 may be a multifunctional ROS-generating enzyme whose activity is predominantly controlled by the presence or absence of GSSG, the native electron acceptor. A similar characterization has also been proposed for human GR (Korge et al., 2015). Indeed, given the reports of ROS production by multiple GRs (Asada, 1999; Coppo et al., 2022; Korge et al., 2015; Liochev and Fridovich, 1992; Massey et al., 1969) and the high sequence similarity of several prototypical GRs to ToGR (Diaz et al., 2019), NOX-like ROS production by GR should be considered in organisms that are taxonomically widespread beyond *T. oceanica.*

GR-mediated ROS production would not necessarily rule out a role for NOX. For example, the *T. oceanica* genome encodes several putative NOX homologs (Diaz et al., 2019), suggesting the coexistence of at least two broad mechanisms for extracellular superoxide production in the same microorganism. This metabolic versatility may provide an adaptive advantage. For instance, NOX proteins are heme-dependent enzymes with a high iron requirement, whereas GR does not depend on iron or any other metal. Iron scarcity constrains the growth and productivity of marine phytoplankton across wide areas of the ocean (Moore et al., 2013), and *T. oceanica* shows a remarkable degree of metabolic flexibility in dealing with chronically low levels of iron (Lommer et al., 2012). We speculate that the substitution of NOX by GR as a source of ROS production may furnish a low-iron benefit to *T. oceanica*, which may also be relevant to other organisms due to the broad role of iron in diverse biological processes. This metal-dependent regulation could potentially help explain why GR would switch from its canonical antioxidant function to a paradoxically pro-oxidant, ROS-generating role.

### Limitations of the study

This study presents enzyme activity results from pure enzymes *in vitro*, which provide insights into the potential activity of these enzymes *in vivo*, as discussed above.

## STAR★Methods

### Key resources table

**Table:** 

REAGENT or RESOURCE	SOURCE	IDENTIFIER
**Bacterial and virus strains**		

*E. coli* BL21 (DE3)	Sigma	CMC0014-4X40UL

**Chemicals, peptides, and recombinant proteins**		

IPTG	Teknova	I3325
Imidazole	Sigma	792527-500G
Glutathione disulfide	Sigma	G4376-500MG
NADPH	Roche	10107824001
DTNB	Sigma	D8130-500MG
Nitroblue tetrazolium	Fisher	BP1081
Superoxide dismutase	Sigma	S5395-30KU
Diphenyleneiodonium	Sigma	D2926-10mg
*T. oceanica* glutathione reductase (ToGR1)	This study	N/A
*T. oceanica* glutathione reductase (ToGR2)	This study	N/A

**Critical commercial assays**		

Q5 site-directed mutagenesis kit	New England Biolabs	E0552
Coomassie (Bradford) kit	ThermoFisher	23200

**Recombinant DNA**		

ToGR2 gene	Twist Bioscience	GenBank AGNL01048094.1

**Software and algorithms**		

Origin Pro (9.9)	OriginLab	N/A
JMP Pro (16.0)	SAS	N/A

**Other**		

pET21 vector	Twist Bioscience	n/a
HisPur Ni-NTA resin	ThermoFisher	88221
Polyacrylamide gels	BioRad	4561036
Amicon Ultra centrifugal filter	Millipore Sigma	UFC801024

### Resource availability

#### Lead contact

Further information and requests for resources and reagents should be directed to and will be fulfilled by the lead contact, Julia Diaz (j2diaz@ucsd.edu).

#### Materials availability

This study did not generate new unique reagents.

### Method details

#### Cloning, protein expression, and purification

The ToGR2 gene (GenBank AGNL01048094.1) was synthesized by Twist Bioscience after intron removal and codon optimization for expression in *E. coli*. The gene was subcloned into pET21 vector with C-terminal His-tag. The ribosome binding site was inserted upstream of the gene. To remove the putative N-terminal localization domain of ToGR2, N-terminal truncated ToGR2 (NT-ToGR2) was created by deleting 130 amino acids at the N-terminus using the Q5 site-directed mutagenesis kit (New England BioLabs). NT-ToGR1 was created from NT-ToGR2 by replacing three amino acids, Asn248Asp, Lys325Ile, and Asp480Glu, consistent with previous sequence analysis (Diaz et al., 2019).

*E. coli* BL21 (DE3) cells were used to overexpress NT-ToGR1 and NT-ToGR2 (hereafter ToGR1 and ToGR2). Briefly, cells were grown at 37°C to an optical density (at 600 nm) of 0.5–0.8, and chilled to 25°C. Protein overexpression was induced with 0.1 mM IPTG, and the cells were grown for another 3 h. To purify ToGR1 and ToGR2, the cell pellet was resuspended in buffer A (50 mM Tris-HCl pH 8, 200 mM NaCl, 10% glycerol) with 10 mM imidazole and lysed using a French press at 18000 psi (lysate). The lysate was centrifuged (15,000×g, 50 min, 4°C) to isolate the soluble proteins (supernatant), which were then purified by Ni-NTA affinity chromatography (ThermoFisher) following the manufacturer’s instructions. Briefly, the sample flow-through was discarded, the column was washed with buffer A containing 20 mM imidazole, and the target proteins were eluted by 300 mM imidazole in buffer A. Protein fractions were analyzed with SDS-PAGE (180 V, 30 min) using 10% pre-cast polyacrylamide gels (Bio-Rad) and stained with Coomassie blue. Purified ToGR1 and ToGR2 proteins were stored at −80°C in buffer containing 50 mM Tris-HCl, pH 7.5, 150 mM NaCl, 1 mM DTT, and 10% glycerol. Protein concentrations were measured using the Bradford method (ThermoFisher).

#### Protein spectroscopy

Protein absorption spectra were recorded on a SpectraMax M3 plate reader (Molecular Devices) at 25°C in phosphate buffer (100 mM potassium phosphate and 1 mM EDTA, pH 8.0) or Tris buffer (100 mM Tris-HCl and 1 mM EDTA at pH 8.0) using 10 μM of purified ToGR1 or ToGR2. Proteins were fully reduced with 0.1 mM DTT or fully oxidized with 1 mM of GSSG. Additional spectra were collected under conditions of superoxide production in the presence of NADPH (120 μM) and in the absence of GSSG. All protein spectra were blank-corrected using enzyme-free controls. Peak positions were determined using the Quick Peaks Gadget in Origin Pro (9.9) with default settings.

#### Enzyme activity assays

All reactions were carried out in 200 μL reaction volume in a clear, flat-bottom 96-well plate at 25°C on a SpectraMax M3 plate reader (Molecular Devices) in the presence or absence of ToGR1 or ToGR2. Enzyme-free controls were used to determine uncatalyzed reaction rates. All concentrations indicated are final.

##### Glutathione reductase native activity

ToGR-mediated GSSG reduction and NADPH oxidation were measured using the DTNB assay. In this assay, GR reduces GSSG to GSH, which reacts with 5,5′-Dithio-*bis*-(2-nitrobenzoic acid) (DTNB) to produce the yellow product 2-nitro-5-thiobenzoic acid (TNB). Kinetic analyses were performed in phosphate buffer (100 mM potassium phosphate, 1 mM EDTA, pH 8.0) by varying the concentration of NADPH (0-80 μM) at fixed concentrations of GSSG (2 mM) and DTNB (0.1 mM). The reaction was started by the addition of ToGR1 or ToGR2 (0.045 nM, final enzyme concentration). Absorbance measurements were taken at 412 and 340 nm every 1.5 min for 1 h to monitor TNB production, and NADPH oxidation, respectively.

To determine linear reaction rates (R^2^ > 0.98), each absorbance value was first blank-corrected for controls containing all components except NADPH. Next, linear reaction rates (abs min^−1^) were converted to molar units (nmol L^−1^ min^−1^) by applying the molar extinction coefficient for TNB (14.15 mM^−1^ cm^−1^) (Eyer et al., 2003) or NADPH (6.22 mM^−1^ cm^−1^). Finally, these rates were corrected by subtracting enzyme-free controls. GSH production rates were calculated assuming 1:1 stoichiometry of TNB:GSH (Smith et al., 1988).

##### Superoxide production

ToGR-mediated superoxide production and NADPH oxidation were measured using the NBT assay. In this assay, superoxide reacts with nitroblue tetrazolium (NBT) to produce the purple product monoformazan (MF^+^). The amount of superoxide produced by GR is proportional to the amount of MF^+^ production that is inhibited by the enzyme superoxide dismutase (SOD). Kinetic analyses were performed at pH 8.0 in phosphate buffer (100 mM potassium phosphate, 1 mM EDTA) or Tris buffer (100 mM Tris-HCl, 1 mM EDTA) in the presence of 0.1 mM NBT with or without the addition of SOD (200 U mL^−1^) by varying the concentration of NADPH from 0-300 μM (ToGR1) or 0-600 μM (ToGR2). Reactions were assumed to be in equilibrium with atmospheric oxygen levels. Unless otherwise stated, reactions were started by the addition of ToGR1 (40 nM) or ToGR2 (200 nM). Absorbance measurements were taken at 530 and 340 nm every 1.5 min for 1 h to monitor MF^+^ production, and NADPH oxidation, respectively.

Several types of inhibition experiments were carried out with the following modifications to the NBT assay. To test the effect of DPI, ToGR1 (40 nM) or ToGR2 (400 nM) was incubated with DPI (0-50 μM in 0.5% DMSO) for 10 min at room temperature, and reactions were started in the presence of DPI by the addition of 200 μM NADPH (final concentrations). To test the effect of formaldehyde, ToGR1 and ToGR2 were pre-treated with formaldehyde (see below), which was removed before beginning the NBT assay containing 200 μM NADPH and 40 nM ToGR1 or 400 nM ToGR2 (final concentrations). In select inhibition experiments, GSSG was added to 20 μM.

To determine rates of MF^+^ and superoxide production, the absorbance of MF^+^ was first blank-corrected for controls containing all components except NADPH. To quantify superoxide production rates, these measurements were further corrected by subtracting SOD controls. Rates of MF^+^ production (abs min^−1^) were determined over the linear range (R^2^ > 0.98) and converted to molar units (nmol L^−1^ min^−1^) by applying the molar extinction coefficient of MF^+^ (20 mM^−1^ cm^−1^) (Bielski et al., 1980). These rates were converted to superoxide production using the O_2_^−^:MF^+^ reaction stoichiometry of 2:1(Bielski et al., 1980). Finally, reaction rates were corrected by subtracting enzyme-free controls. Rates of NADPH oxidation were determined as above for the DTNB assay. Reaction stoichiometries were determined from multiple observations of NADPH, MF^+^, and superoxide concentrations across the reaction time course.

#### Formaldehyde treatment of ToGR

ToGR1 and ToGR2 were treated with formaldehyde as described previously (Diaz et al., 2019). Briefly, proteins were incubated at 4°C for 2 h in Tris buffer (100 mM Tris-HCl, 1 mM EDTA, pH 8.0) amended with 0% (control), 1.5%, or 4% formaldehyde (final concentrations). Formaldehyde was removed by changing into Tris buffer using a 10 kDa Amicon Ultra-0.5 centrifugal filter device (Millipore Sigma), according to the manufacturer’s instructions. Formaldehyde-treated proteins were incubated for an hour at 4°C prior to analysis with the NBT assay.

#### Numerical modeling

Catalyzed rates (*R*) of NADPH oxidation, GSH production, and superoxide production were fit to the following Michaelis-Menten equation by minimizing the sum of squared residuals using the Solver tool in Microsoft Excel:

(Equation 1) $$R=\frac{k_{cat}{\left[E\right]}_{t}\left[S\right]}{K_{m}+\left[S\right]}$$

where [*S*] is the concentration of NADPH, [*E*]_t_ is the concentration of enzyme, and the fitted parameters are the enzyme turnover number (*k*_*cat*_) and half saturation constant (*K*_*m*_). Uncatalyzed reaction rates were determined as the log-linear (R^2^ > 0.9) pseudo first-order rate constant of the enzyme-free controls (Table S2).

To quantify DPI inhibition, IC_50_ values were calculated from the linear regression of log-transformed DPI concentrations versus the percent inhibition of NADPH oxidation or superoxide production (R^2^ >0.94). IC_50_ values were calculated according to the formula: IC_50_ = (0.5-*b*)/*a*, where *b* is the y-intercept and *a* is the slope of the linear regression.

### Quantification and statistical analysis

Statistical analyses were performed in JMP Pro (16.0). Reaction rates and stoichiometries were compared using Student’s *t* test or Tukey’s Honest Significant Difference (HSD) test, and p values <0.05 were considered significantly different.

## Data Availability

• All data reported in this paper will be shared by the lead contact upon request. • This paper does not report original code. • Any additional information required to reanalyze the data reported in this paper is available from the lead contact upon request.
