# Supplementary material for: NOX-like ROS production by glutathione reductase
Source: iScience. 2022 Sep 8;25(10):105093. doi: 10.1016/j.isci.2022.105093 (PMC9519596; doi:10.1016/j.isci.2022.105093)
Supplement: Document S1. Figures S1–S4 and Tables S1 — and S2 [file mmc1.pdf]

iScience, Volume *25*

Supplemental information

NOX-like ROS production by glutathione reductase

Julia M. Diaz and Xinying Shi

Table S1. IC_{50} values of DPI inhibition, related to Fig 4c and Fig S3

Enzyme	Substrate or product	IC_{50} (μM)
ToGR1	NADPH	36.4
	O_2^-	7.9
ToGR2	NADPH	1831
	O_2^-	545

Table S2. Uncatalyzed rate constants, related to Table 1 and STAR methods
Data represent the avg. ± std. dev. of 22 observations

Substrate or product	k_{uncat} (min^{-1})	n
NADPH	$(2.3\pm0.9)\times10^{-4}$	22
GSH	$(1.2\pm0.0)\times10^{-3}$	22
O_2^-	$(8.4\pm5.7)\times10^{-4}$	22

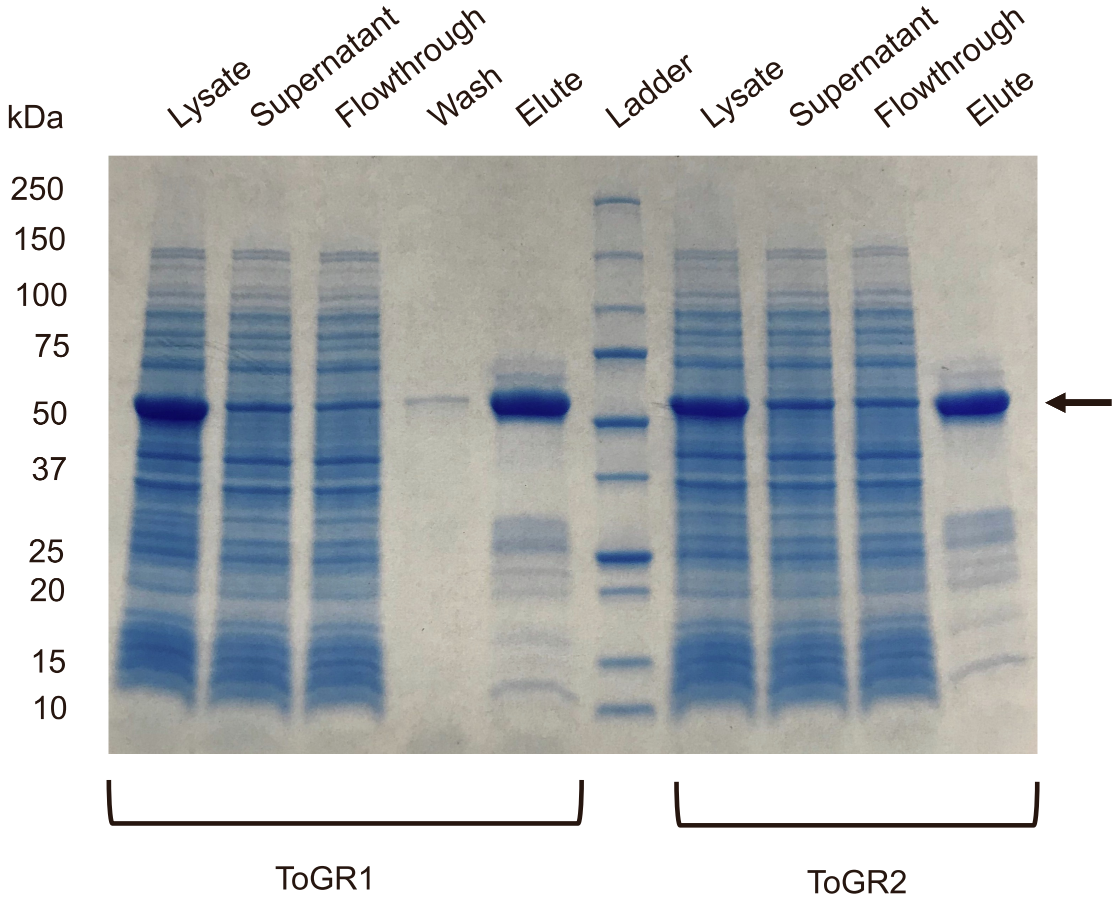

Fig S1. SDS-PAGE analysis of protein fractions obtained during the purification of recombinant ToGR1 and ToGR2, related to Fig 1a and STAR methods. Arrow indicates ~55 kDa

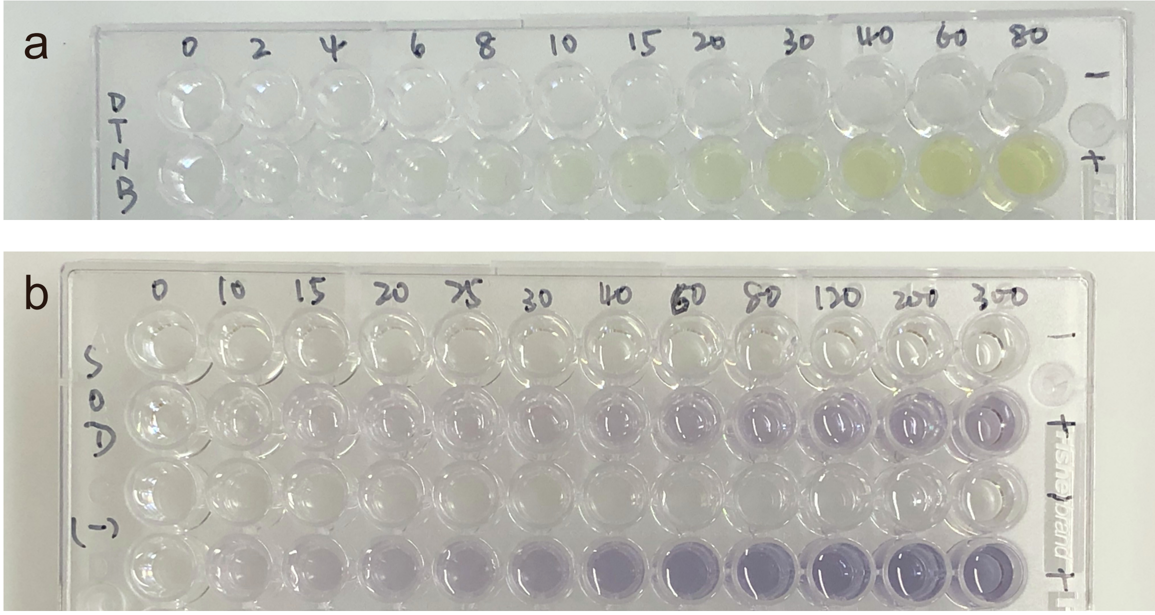

Fig S2. Activity assays showing color changes, related to Fig 1d,e and STAR methods. GSSG reduction (a) or superoxide production (b) by ToGR1. Top: NADPH concentration (μM); Left: presence or absence (-) of SOD, as indicated; Right: presence (+) or absence (-) of ToGR1

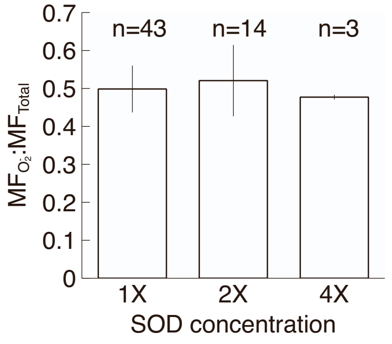

Fig S3. Proportion of superoxide-mediated monoformazan (MF) production by ToGR1 in the presence of increasing concentrations of superoxide dismutase (SOD), related to Fig 2c-f. 1X = 200 U mL^{-1}. Data represent the avg. ± std. dev. of n observations indicated above each bar. Results were statistically similar across all levels of SOD ($p \geq 0.55$, Tukey HSD)

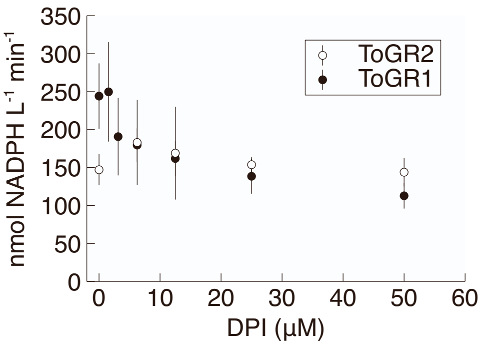

Fig S4. NADPH oxidation by ToGR1 and ToGR2 in the presence of DPI, related to Fig 4c. Data represent the avg. ± std. dev. of triplicate observations
